# Supplementary material for: The Effect of Postinduction Blood Glucose on Intraoperative Hypothermia
Source: Medicina (Kaunas). 2023 Feb 17;59(2):395. doi: 10.3390/medicina59020395 (PMC9959156; doi:10.3390/medicina59020395)
Supplement: Supplementary file 1 [file medicina-59-00395-s001.zip › medicina-2178566-supplementary.pdf]

Table S1. Patient characteristics for hypothermic and normothermic patients.

	Hypothermic (n = 200)	Normothermic (n = 134)	p Value
Comorbidities n (%)			
Diabetes	72 (36.0)	49 (36.6)	0.916
Hypertension	19 (9.5)	7 (5.2)	0.153
Cardiac disease	8 (4.0)	7 (5.2)	0.409
Cerebrovascular disease	1 (0.5)	0 (0.0)	0.412
Chronic Obstructive Pulmonary Disease	1 (0.5)	1 (0.1)	0.775
Types of surgery n (%)			
Esophageal surgery	39 (19.5)	21 (15.7)	0.372
Gastric surgery	59 (29.5)	38 (28.3)	0.822
Intestinal surgery	78 (39.0)	51 (38.1)	0.863
Hepatopancreatobiliary surgery	24 (12.0)	24 (17.9)	0.131
Types of anesthesia n (%)			
Total intravenous anesthesia	26 (13.0)	11 (8.2)	0.172
Volatile anesthesia	43 (21.5)	25 (18.6)	0.527
Combined epidural and total intravenous anesthesia	36 (18.0)	23 (17.2)	0.844
Combined epidural and volatile anesthesia	95 (47.5)	75 (56.0)	0.129

Data are shown as frequencies n (%).
